# Comparative associations of the TyG index and HOMA2-IR with 6-month outcomes after moderate-to-severe traumatic brain injury: a single-center retrospective cohort study

**DOI:** 10.3389/fnmol.2026.1826985

**Published:** 2026-06-24

**Authors:** Hai Zhao, Lingjuan Gao, Wei Wu, Cheng Cao, Heng Gao

**Affiliations:** 1 Department of Emergency Medicine, Jiangyin Clinical College of Xuzhou Medical University, Jiangyin, Jiangsu, China; 2 Department of Neurosurgery, Jiangyin Clinical College of Xuzhou Medical University, Jiangyin, Jiangsu, China; 3 Department of Brain Center, Jiangyin Clinical College of Xuzhou Medical University, Jiangyin, Jiangsu, China; 4 Department of Intensive Care Unit, Jiangyin Clinical College of Xuzhou Medical University, Jiangyin, Jiangsu, China

**Keywords:** homeostasis model assessment of insulin resistance, insulin resistance, moderate-to-severe traumatic brain injury, prognosis, triglyceride glucose index

## Abstract

**Background:**

The triglyceride glucose (TyG) index and the homeostatic model assessment 2 of insulin resistance (HOMA2-IR) are both valid markers for assessing insulin resistance (IR) and may serve as early metabolic biomarkers associated with outcomes in patients with moderate-to-severe traumatic brain injury (msTBI). This study was designed to directly compare the TyG index and HOMA2-IR regarding their associations with 6-month outcomes in msTBI patients.

**Methods:**

This study conducted a single-center, retrospective cohort of 362 consecutive patients with msTBI admitted to the Jiangyin Clinical College of Xuzhou Medical University. Six-month outcomes were evaluated using the Glasgow Outcome Scale–Extended (GOSE) and dichotomized as favorable (GOSE ≥ 5) or unfavorable (GOSE ≤ 4). In this study core IMPACT-CT variables were collected (Glasgow Coma Scale score, age, pupillary reactivity, and Marshall CT score), sex, and two IR indices. Analyses included univariable comparisons by the outcome group, evaluation of the correlation between the TyG index and HOMA2-IR, generalized additive models (GAMs) to assess potential non-linearity, and multivariable models with adjusted restricted cubic splines (RCSs) to confirm and characterize non-linear associations of the TyG index and HOMA2-IR with outcome and to clarify their independent contributions. Clinical utility was evaluated using decision curve analysis (DCA) to determine net benefit.

**Results:**

All core IMPACT-CT variables and both IR indices differed significantly by the outcome group. The TyG index and HOMA2-IR were moderately correlated. GAMs indicated a mildly non-linear negative association between the TyG index and an approximately linear negative association between HOMA2-IR and the outcome. RCSs confirmed that both indices were strongly associated with outcome and similarly improved discrimination, whereas the TyG index showed greater independent contribution and a broader range of net benefit on DCA.

**Conclusion:**

Both the TyG index and HOMA2-IR were significantly associated with unfavorable outcomes and improved the performance of models based on core IMPACT-CT variables in this single-center retrospective cohort study. Although their overall discrimination was similar, the TyG index showed a modest but consistent advantage in independent contribution and net clinical benefit, and it was easier to implement in clinical practice.

## Introduction

Moderate-to-severe traumatic brain injury (msTBI) is a major global cause of neurological disability and death (Gao et al., 2020; McCrea et al., 2021). Its pathophysiology is complex and involves both primary and secondary injuries, including ongoing neuroinflammation, disruption of the blood–brain barrier, and systemic metabolic dysregulation (Visser et al., 2022). These metabolic dysregulations include abnormal glucose metabolism, lipid metabolism disorders, and electrolyte imbalances (Lai et al., 2022; Venturini et al., 2023; Ngatuvai et al., 2023). Insulin resistance (IR) is an important contributor to systemic metabolic dysregulation after msTBI and is closely linked to post-injury glucose abnormalities (Cao et al., 2022; Shpakov et al., 2023).

IR refers to reduced tissue sensitivity to insulin, leading to impaired insulin action and abnormal glucose metabolism, resulting in systemic hyperglycemia that can drive disease progression (Yang et al., 2024; Hajikarimloo et al., 2025). Prior studies have consistently shown strong associations between IR and adverse outcomes across multiple diseases, including cardiovascular disease, kidney disease, thyroid disorders, and Alzheimer’s disease (Safari et al., 2024; Xie et al., 2025; Yue et al., 2025; Sun and Mi, 2025). Several indicators can be used to assess IR, such as the triglyceride glucose (TyG) index, the fasting glucose-to-insulin ratio, and the Homeostasis Model Assessment (HOMA)-based indices, including the original homeostatic model assessment of insulin resistance (HOMA-IR), and the updated computer-based homeostatic model assessment 2 of insulin resistance (HOMA2-IR) (Matthews et al., 1985; Tahapary et al., 2022; Ma et al., 2024). Among these, the TyG index and HOMA-based indices are widely used. The TyG index is considered practical because it relies on readily available and low-cost laboratory parameters (Gastaldelli, 2022).

Earlier studies by the group and others have shown that IR is a risk factor for unfavorable outcomes in patients with msTBI (Cao et al., 2022; Mowery et al., 2009; Cao et al., 2024). However, the relative prognostic performance of two commonly used IR indices, the TyG index and HOMA2-IR, remains unclear in msTBI. To address this gap, a retrospective cohort study of 362 msTBI patients admitted to the Jiangyin Clinical College of Xuzhou Medical University was conducted, comparing the predictive value of the TyG index and HOMA2-IR for patient outcomes. This study was designed to directly compare the TyG index and HOMA2-IR regarding their discriminatory power for predicting poor outcomes in msTBI patients, thereby enabling earlier identification of patients at high risk due to IR and providing a basis for future IR-targeted clinical interventions.

## Methods

### Study design and ethics

A single-center retrospective cohort study was performed at the Department of Neurocritical Care, Jiangyin Clinical College of Xuzhou Medical University. This study was conducted according to an ethics-approved retrospective observational protocol approved by the institutional Ethics Committee of Jiangyin Clinical College of Xuzhou Medical University (Approval No. 2020053), and it complied with the Declaration of Helsinki. As this was a retrospective study using de-identified routinely collected clinical data and involving no assignment of interventions, informed consent was waived, and the study was not prospectively registered in a public trial registry. To enhance transparency, the TRIPOD-AI checklist was completed and provided as Supplementary Table S1.

### Study population

Consecutive adults with msTBI admitted within 24 h of injury from January 2019 to December 2023 were screened. Inclusion criteria were: (1) age ≥ 18 years, (2) confirmed TBI by CT, and (3) Glasgow Coma Scale (GCS) during admission ≤ 12. Exclusion criteria included: (1) major extracranial injury Abbreviated Injury Scale > 1 outside the head; (2) severe chronic comorbidities or malignancies likely to affect short-term survival; and (3) missing core variables, IR indices, or 6-month outcome.

### Data collection

Within 24 h of admission, fasting triglycerides, fasting blood glucose, and fasting insulin were obtained according to institutional protocols. Specifically, the first fasting venous blood sample was obtained within 24 h after admission. All patients with msTBI were maintained on NPO status throughout the first 24 h after admission. Routine blood sampling was scheduled between 5:00 and 6:00 a.m. If the patient was confirmed to be fasting at this time point, blood samples were collected immediately. If fasting status was unknown, sampling was postponed until fasting status could be reasonably confirmed, generally after an additional 6–8 h of observation, including the waiting time in the emergency department before admission. Sex and core IMPACT-CT variables were recorded within 24 h, including GCS, age, pupillary reactivity, and the worst Marshall CT score.

### Insulin resistance indices

The TyG index was calculated as ln [fasting triglycerides (mg/dL) × fasting glucose (mg/dL)/2]. HOMA2-IR was computed using the HOMA2 Calculator (https://www.dtu.ox.ac.uk, University of Oxford, version 2.2.3) with accepted input ranges for glucose and insulin. Patients with glucose or insulin values outside the accepted input range of the HOMA2 calculator were considered to have unacceptable values and were excluded because HOMA2-IR could not be computed.

### Outcomes

Six-month outcomes were assessed using the Glasgow Outcome Scale–Extended (GOSE) by clinic visit or structured telephone interview and dichotomized as unfavorable (GOSE ≤ 4) or favorable (GOSE ≥ 5).

### Study sample size

A sample size calculation was performed during the study protocol design stage. The primary outcome was an unfavorable 6-month functional outcome. Based on historical data from our departmental traumatic brain injury registry, the expected unfavorable 6-month outcome rate among patients with msTBI was approximately 40%. For the planned multivariable logistic regression analyses, a maximum of 13 candidate predictor parameters, including the core IMPACT-CT variables and the two IR indices were anticipated. Based on the conventional requirement of at least 10 outcome events per predictor parameter, a minimum of 130 unfavorable outcome events was required. Therefore, with an expected unfavorable outcome rate of 40%, the minimum required sample size was estimated to be 325 patients. Finally, 362 consecutive patients met the eligibility criteria and were included in the study cohort, exceeding the prespecified minimum sample size.

### Statistical analysis

The study compared baseline variables by the outcome group and estimated the correlation between TyG index and HOMA2-IR. The adjusted GAMs were used to assess potential non-linear associations between each IR index and unfavorable outcome. Logistic models with RCSs were fitted to characterize functional forms and estimate adjusted effects, and evaluated model performance in terms of discrimination using receiver operating characteristic(ROC)curves and area under the curve(AUC) with DeLong confidence intervals, calibration using the Brier score and calibration plots, parsimony using Akaike Information Criterion (AIC) and Corrected AIC (AICc), independent contribution using drop-one likelihood ratio tests and changes in AICc, internal validity using bootstrap optimism correction, and clinical utility using DCA across clinically relevant thresholds. Continuous variables are reported as mean (SD) or median (IQR) and compared using *t*-tests or Mann–Whitney *U*-tests. Categorical variables are presented as counts (%) and compared using chi-square or Fisher’s exact tests. All statistical analyses were performed using R software (Version 4.5.2, R Foundation for Statistical Computing). A two-tailed *p*-value of < 0.05 was considered statistically significant.

## Results

### Univariable analyses by outcome groups

Compared with patients with favorable outcomes, those with unfavorable outcomes were older (median age 60.0 vs. 53.0 years, *p* < 0.001) and had lower admission GCS scores (median 4 vs. 9, *p* < 0.001). Pupillary reactivity and Marshall CT score differed markedly between groups (both *p* < 0.001), non-reactive pupils and higher Marshall grades were more frequent among unfavorable outcomes. Sex distribution did not differ between the groups (*p* = 0.41). Both IR indices were higher in the unfavorable group: TyG index 8.87 vs. 8.46 (*W* = 10380.0, *p* < 0.001) and HOMA2-IR 3.04 vs. 1.74 (*W* = 9644.5, *p* < 0.001). Unfavorable outcomes were associated with both a higher TyG index (8.87 vs. 8.46, *p* < 0.001) and a higher HOMA2-IR (3.04 vs. 1.74, *p* < 0.001) (Table 1).

**Table 1:** Univariable analyses by outcome.

Variable	Favorable outcome	Unfavorable outcome	Statistic	*P*-value
AGE (IQR)	53.00 [43.50, 65.00]	60.00 [50.00, 68.00]	*W* = 12318.0	<0. 001
GCS (IQR)	9 [7, 11]	4 [3, 7]	*W* = 25088.0	<0. 001
Gender				
Male	48 (22.7%)	40 (26.5%)	*Χ*^2^ = 0.670	0.41
Female	163 (77.3%)	111 (73.5%)		
Pupillary reactivity				
No pupils reacted	41 (19.4%)	91 (60.3%)	*W* = 24160.5	<0. 001
One pupil reacted	36 (17.1%)	33 (21.9%)		
Both pupils reacted	134 (63.5%)	27 (17.9%)		
Marshall CT score				
I/II	35 (16.6%)	2 (1.3%)	*W* = 10304.0	<0. 001
III/IV	94 (44.5%)	48 (31.8%)		
V	74 (35.1%)	81 (53.6%)		
VI‌	8 (3.8%)	20 (13.2%)		
TyG Index (IQR)	8.46 [7.97, 8.89]	8.87 [8.42, 9.46]	*W* = 10380.0	<0. 001
HOMA2-IR (IQR)	1.74 [1.27, 2.88]	3.04 [1.87, 4.83]	*W* = 9644.5	<0. 001

### Correlation between TyG index and HOMA2-IR

**Figure 1:**
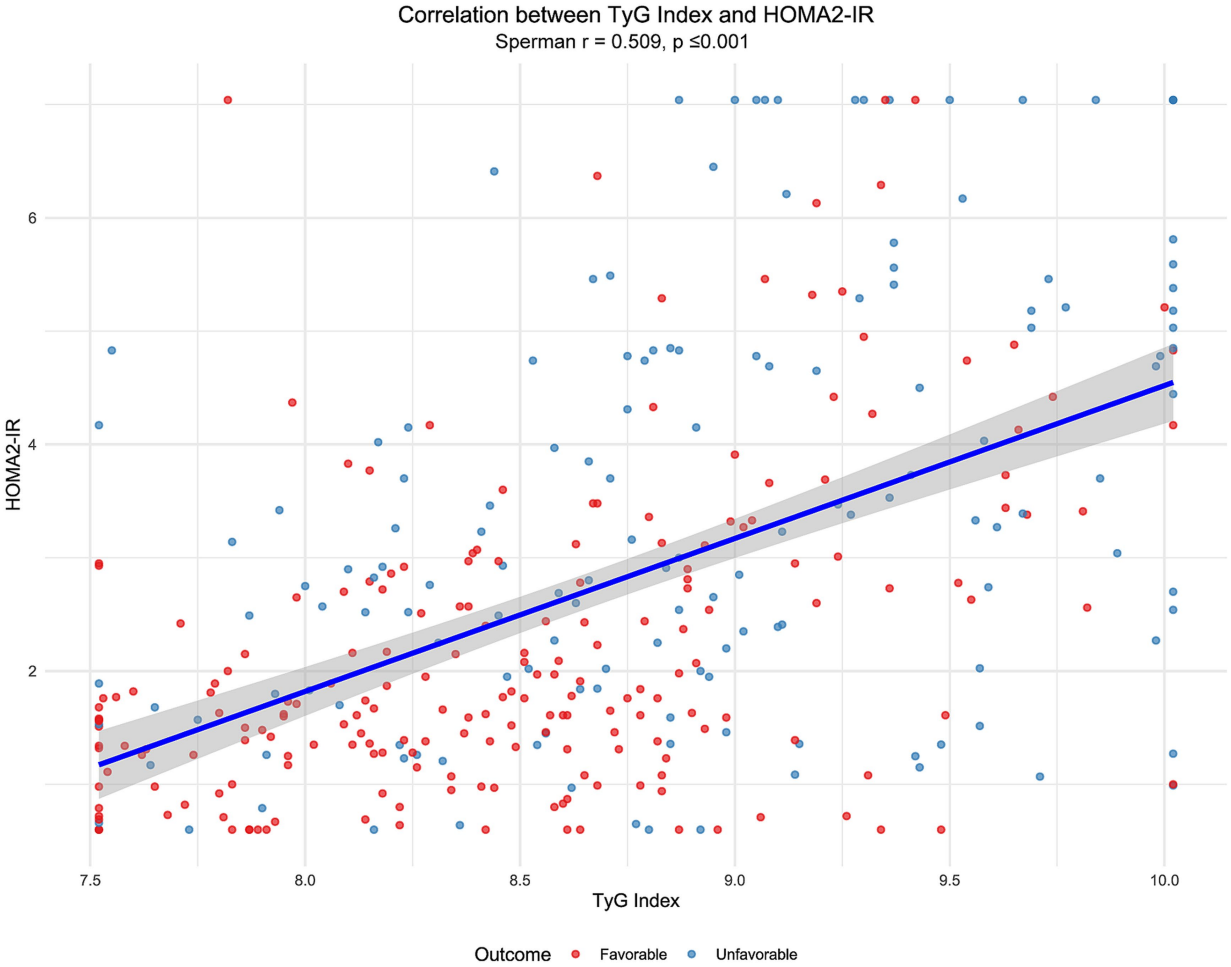
Correlation between the TyG index and HOMA2-IR in patients with msTBI. Each dot represented one patient and was colored by outcome group (red, favorable; blue, unfavorable). The solid line depicted the fitted linear trend with a 95% confidence band (gray). Spearman’s correlation coefficient was *r* = 0.509 (*p* < 0.001), indicating a moderate positive association between the two indices.

TyG index and HOMA2-IR were moderately and positively correlated (Spearman’s *r* = 0.509, *p* < 0.001), with higher TyG index values associated with higher HOMA2-IR across the observed range (Figure 1).

### Generalized additive models

**Figure 2:**
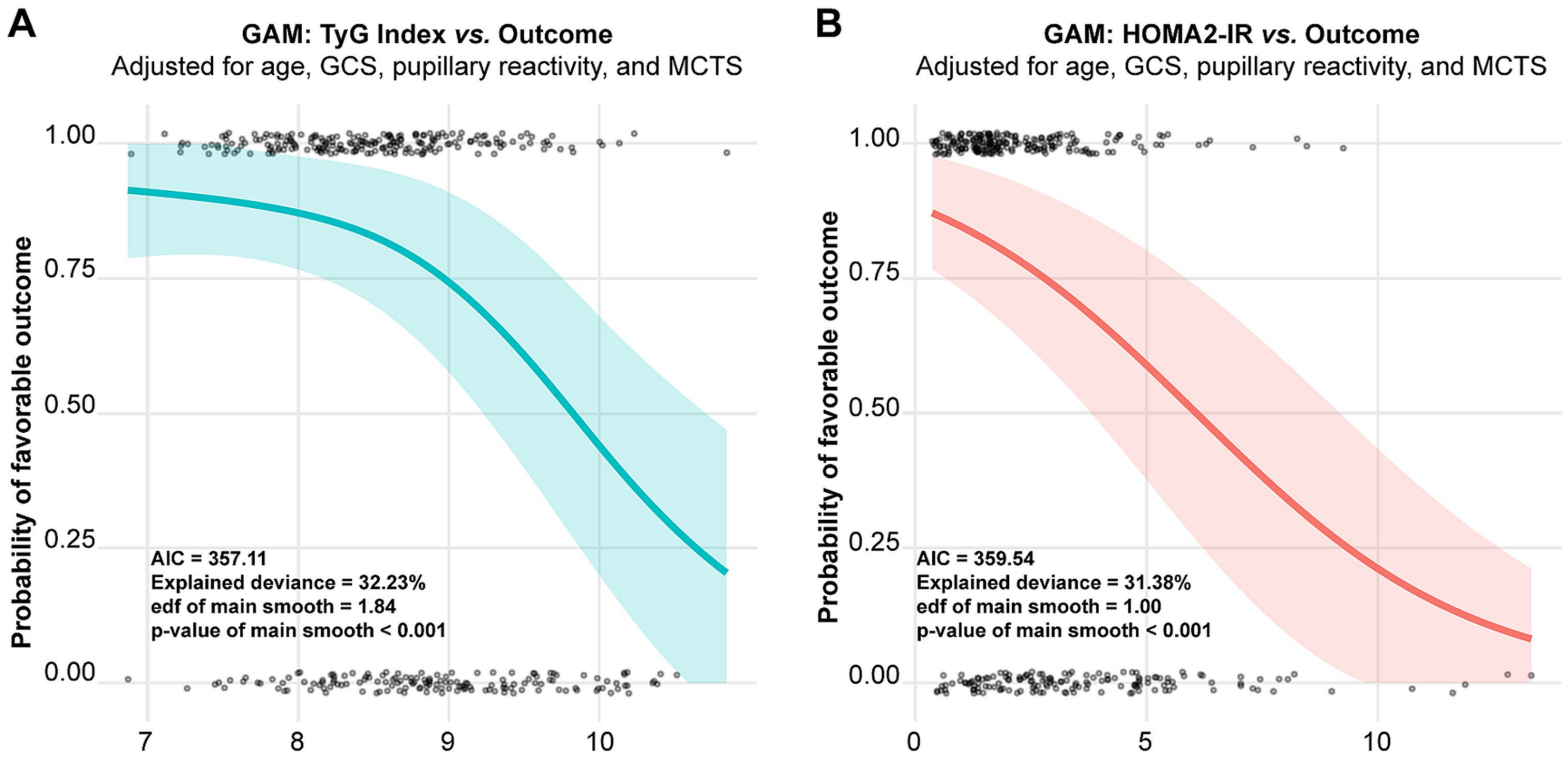
Adjusted GAMs estimated for the association of IR indices with the probability of a favorable outcome. **(A)** TyG index shows a mildly non-linear negative association (edf = 1.84; explained deviance = 32.23%; *p* < 0.001; AIC = 357.11). **(B)** HOMA2-IR exhibits an approximately linear negative association (edf = 1.00; explained deviance = 31.38%; *p* < 0.001; AIC = 359.54). Shaded areas represented 95% confidence bands; tick marks along the x-axis indicated observed values.

Given that all core IMPACT-CT variables differed significantly between outcome groups in univariable analyses, four covariates were adjusted in the GAMs (age, GCS, pupillary reactivity, Marshall CT score). After adjustment, both indices showed negative relationships with the probability of a favorable outcome (Figure 2). The TyG index effect displayed mild curvature (edf 1.84), consistent with a near-monotonic but slightly non-linear decline in favorable outcome as the TyG index increased (*p* < 0.001; explained deviance 32.23%; AIC 357.11). For HOMA2-IR, the relationship was essentially linear on the logit scale (edf 1.00) with higher values associated with lower chances of a favorable outcome (*p* < 0.001; explained deviance 31.38%; AIC 359.54).

### Restricted cubic spline models

**Figure 3:**
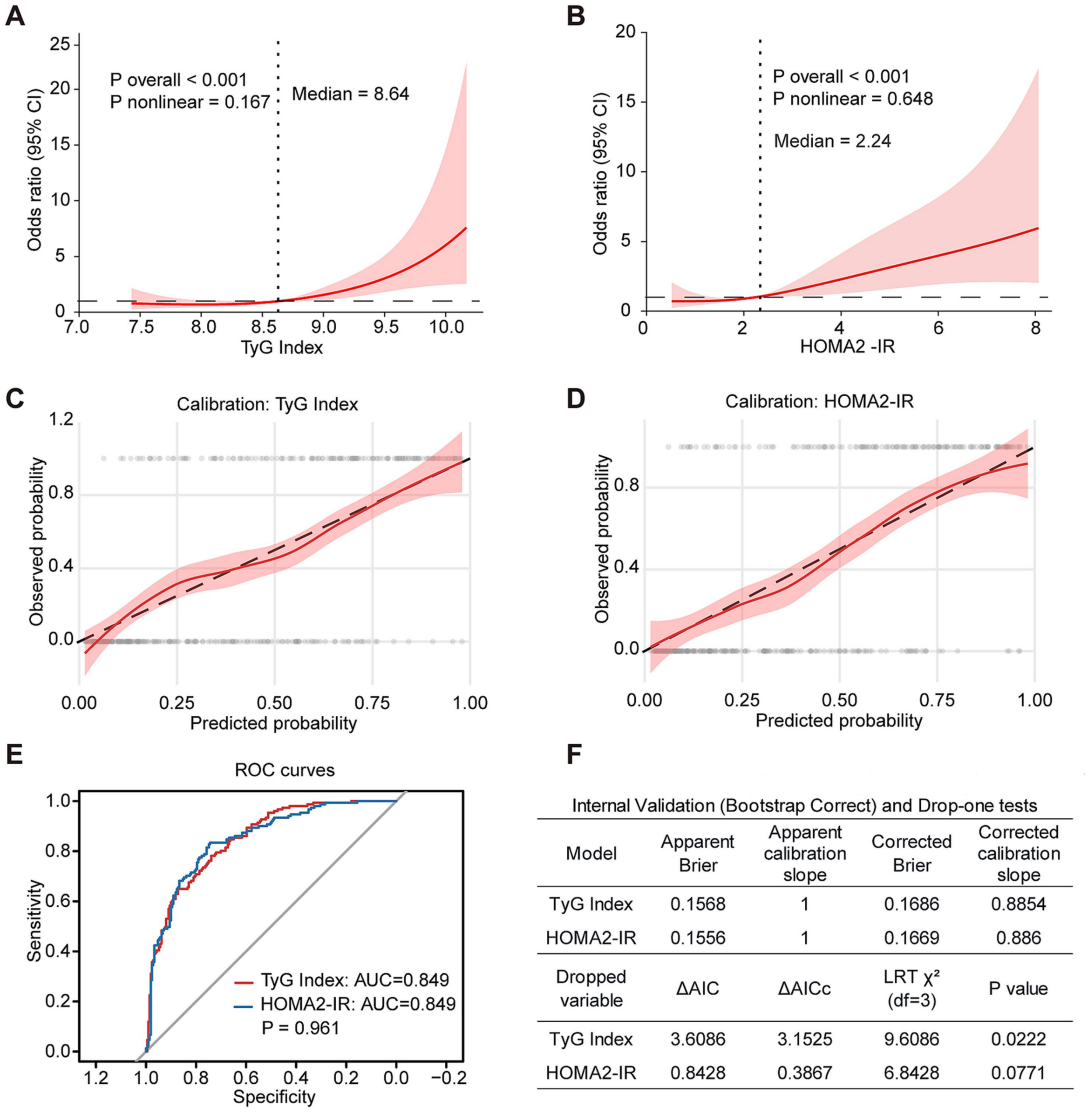
RCS logistic models and model performance. **(A,B)** Adjusted exposure–response curves for the TyG index **(A)** and HOMA2-IR **(B)** were expressed as odds ratios relative to each variable’s median. Overall associations were significant (both *p* < 0.001) with limited evidence of non-linearity (TyG index *P* for non-linearity = 0.167; HOMA2-IR P for non-linearity = 0.648). **(C,D)** Calibration plots compare predicted and observed probabilities for models incorporating the TyG index **(C)** or HOMA2-IR **(D)**; shaded areas indicated 95% confidence bands around the LOESS smooth. **(E)** ROC curves showed comparable discrimination (*p* = 0.961). **(F)** Internal validation and contribution analyses showed optimism-corrected Brier scores of 0.1686 (TyG index) and 0.1669 (HOMA2-IR) with calibration slopes of 0.885 and 0.886, respectively; drop-one tests indicated a stronger independent contribution of the TyG index (ΔAIC = 3.61; ΔAICc = 3.15; likelihood-ratio χ2 = 9.61; *p* = 0.022) than HOMA2-IR (ΔAIC = 0.84; ΔAICc = 0.39; likelihood-ratio χ2 = 6.84; *p* = 0.077).

In multivariable logistic models with RCSs (Figure 3), both the TyG index and HOMA2-IR were strongly associated with the odds of an unfavorable outcome (overall *p* < 0.001 for each). Evidence for non-linearity was not compelling for either index (TyG index P for non-linearity = 0.167; HOMA2-IR P for non-linearity = 0.648), consistent with approximately monotonic increases in risk across their ranges. Model discrimination was high and comparable whether using the TyG index or HOMA2-IR (DeLong *p* = 0.961). Calibration was acceptable for both indices with Brier scores of 0.1686 for the TyG index and 0.1669 for HOMA2-IR after bootstrap optimism correction, and calibration slopes close to 1 (0.885 for the TyG index; 0.886 for HOMA2-IR). The further information criteria supported retaining either index. Drop-one analyses showed that the TyG index contributed independently to model fit (ΔAIC 3.61; ΔAICc 3.15; LRT χ2 9.61; *p* = 0.022), whereas the evidence for HOMA2-IR was weaker (ΔAIC 0.84; ΔAICc 0.39; LRT χ2 6.84; *p* = 0.077).

### Decision curve analysis

**Figure 4:**
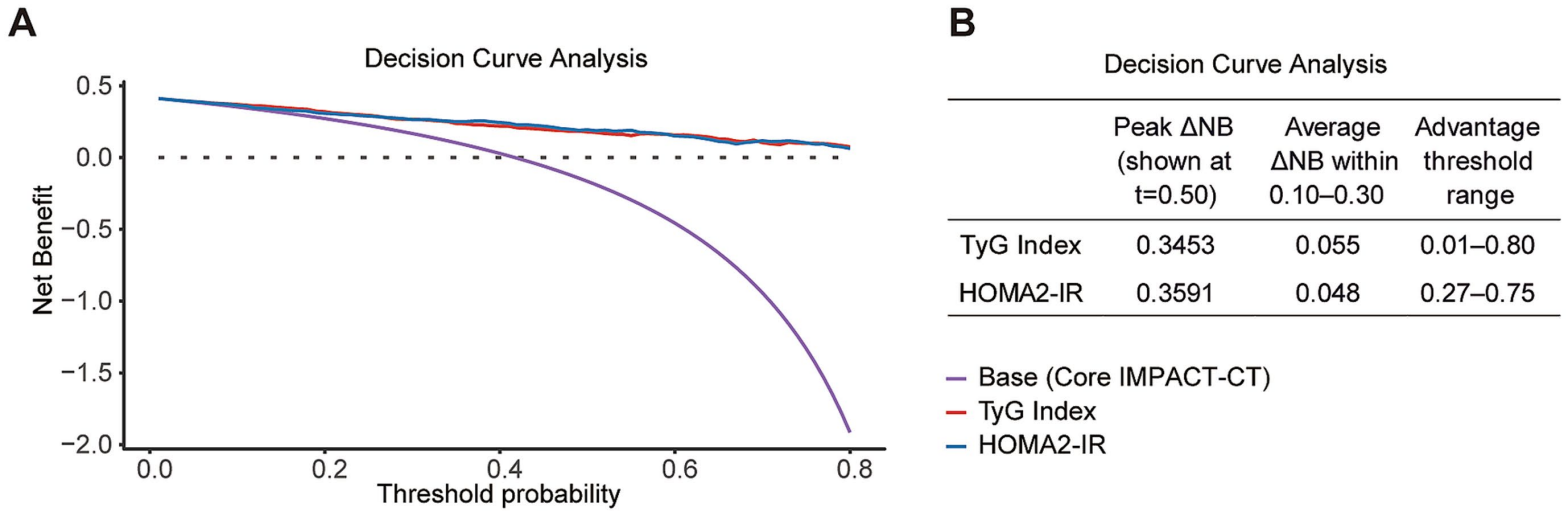
DCA for clinical utility. **(A)** Net benefit across threshold probabilities. **(B)** Summary of net-benefit gains, both augmentations improved clinical utility relative to the base model, with TyG index exhibiting a broader decision-threshold range of advantage. Models: base IMPACT-CT model (age, admission GCS, pupillary reactivity, Marshall CT score), TyG index, and HOMA2-IR (added to the base model and modeled with RCS).

DCA was applied to the final multivariable logistic models, whose predicted probabilities were generated from models that represented TyG index and HOMA2-IR using RCSs (Figure 4). Both augmented models delivered greater net benefit than the base IMPACT-CT model (age, admission GCS, pupillary reactivity, Marshall CT score) across clinically relevant decision thresholds. The TyG index model demonstrated a broader span of advantage, from 0.01 to 0.80, with a peak net-benefit gain at *t* = 0.50 (ΔNB = 0.3453) and an average ΔNB of 0.055 within 0.10–0.30. By comparison, the HOMA2-IR model showed advantages between 0.27 and 0.75, with a peak ΔNB of 0.3591 at *t* = 0.50 and an average ΔNB of 0.048 within 0.10–0.30. Overall, adding either TyG index or HOMA2-IR improved clinical utility compared with the base model, but TyG index contributed more independently and over a wider decision-threshold range.

## Discussion

This study evaluates two IR indices, the TyG index and HOMA2-IR, for their associations with outcomes and their predictive value in patients with msTBI. Higher IR was associated with a lower likelihood of favorable neurological recovery. Evidence from GAMs, RCSs, and DCA suggested that the TyG index and HOMA2-IR have broadly similar overall discrimination. However, the TyG index showed a consistent, modest advantage in independent contribution and the range of probability thresholds with net clinical benefit. These findings indicate that adding metabolic indicators to the core IMPACT-CT variables based on a risk assessment framework can provide incremental value, with the TyG index offering better practical feasibility.

Over two decades, several high-quality clinical studies have moved risk stratification and outcome prediction in msTBI from experience-based practice toward an evidence-based framework (Steyerberg et al., 2008; McCrea et al., 2021; Åkerlund et al., 2024). The IMPACT models and their extensions, including imaging and laboratory variables such as Marshall CT score and glucose, provide a standardized baseline for prognostication and for comparing studies (Steyerberg et al., 2008; Roozenbeek et al., 2012). Several studies linked hyperglycemia and glucose variability with worse neurological outcomes, more infections, and higher mortality, which raised expectations that tighter glycemic control would improve outcomes (Steyerberg et al., 2008; Åkerlund et al., 2024; Qi et al., 2024). Large randomized trials and meta-analyses, conducted with rigorous monitoring and safety protocols, did not show consistent benefits of intensive insulin therapy on mortality or functional outcomes and reported more hypoglycemia, including in the traumatic brain injury (TBI) and neurocritical care subgroups (Finfer et al., 2015; Honarmand et al., 2024; Rivera et al., 2025). The evidence does not challenge the importance of glycemic management; instead, it emphasizes that glycemic targets are set to balance intensity, safety, and individual differences, rather than to pursue uniformly lower targets. A practical approach is to move from uniform intensive glucose lowering to identifying metabolic vulnerability and pursuing individualized control. As a key phenotype of dysregulated glucose and lipid metabolism, IR is not only linked to impaired glucose homeostasis but is also associated with inflammation amplification, mitochondrial dysfunction, disruption of the blood–brain barrier, and impaired perfusion–metabolism coupling, thereby contributing to secondary brain injury after TBI (Gribnau et al., 2024). Our earlier studies (Cao et al., 2022; Cao et al., 2024) and this study all indicated that higher TyG index and HOMA2-IR were associated with unfavorable neurological outcomes. Within the prevailing clinical protocol of glycemic target management, integrating IR assessment into current practice may increase the clinical likelihood of addressing the aforementioned challenges.

Earlier studies indicate that IR is a complex indicator and that its relationship with clinical research endpoints varies across studies, being linear in some contexts and non-linear in others (Wang et al., 2021; Chen et al., 2023; Cai et al., 2023; Song et al., 2023). To reduce model specification bias while preserving interpretability, a two-step strategy was adopted. GAMs were used to screen for potential curvature without prespecifying functional forms and quantified complexity with effective degrees of freedom. Later RCSs were used within logistic regression to parameterize, verify, and compare both indices under the same covariates and assumptions. Augmenting the baseline IMPACT-CT model with either TyG index or HOMA2-IR improved discrimination and calibration, indicating an independent contribution of IR to prognosis. Furthermore, drop-one tests showed more pronounced loss of fit when the TyG index was removed, and DCA indicated a wider range of thresholds with net benefit for the TyG index, which supports its clinical usefulness across diverse decision contexts. These patterns persisted after internal optimism correction. Our findings are consistent with studies in other clinical conditions that have evaluated the utility of the TyG index and HOMA, indicating that the TyG index has demonstrated favorable clinical performance across a range of diseases (Chen et al., 2023; Xiao et al., 2024; Wan et al., 2024; Zhang et al., 2025). However, although this study underscores the value of TyG index, an important issue is highlighted. At high exposure levels, confidence intervals broaden for both indices, consistent with sparse data and greater physiological heterogeneity. Thus, causal inferences at the extremes should be interpreted with appropriate caution.

Beyond statistical performance, the TyG index has advantages that support its clinical adoption. It requires only fasting glucose and triglyceride levels, which are routine in emergency and intensive care settings and have rapid turnaround times suitable for early risk stratification. Unlike insulin assays, which show considerable interlaboratory variability and sensitivity to exogenous insulin, glucose and triglyceride measurements are better standardized and less variable across laboratories (Wallace et al., 2004; Gastaldelli, 2022). These features make the TyG index easier to implement clinically and to use it in multicenter studies.

This study has some limitations. First, it is a single-center cohort with internal validation only, so residual confounding and measurement error are possible. External and temporal validation are still needed, and the causal effects of IR-guided management strategies on clinical outcomes were not evaluated. Future studies should include external validation across multiple centers to assess transportability. Furthermore, in this study, the TyG index and HOMA2-IR were used to characterize the early overall IR status after msTBI, rather than to distinguish the separate contributions of msTBI-related acute metabolic dysfunction and chronic IR. However, pre-existing chronic metabolic conditions, including diabetes, metabolic syndrome, baseline glycemic control, and the use of hypoglycemic medication may also have introduced residual confounding and limited causal interpretation. Future prospective studies should collect and adjust for detailed metabolic history to disentangle the effects of msTBI-related acute metabolic dysfunction from chronic metabolic background. In addition, multimodal integration with imaging, brain biomarkers, and physiological parameters may help test the predictive value of IR across subgroups. Finally, exploring dynamic assessment and individualized threshold strategies will help build continuity of evidence for the clinical application of IR evaluation.

## Conclusion

In conclusion, higher IR was associated with worse outcomes in msTBI. Adding IR indices to the core IMPACT-CT variables improved 6-month prognostic performance in this single-center retrospective cohort study. While overall discrimination was comparable between indices, the TyG index demonstrated a modest, consistent advantage in independent contribution and net clinical benefit, along with greater ease of clinical implementation.

## Data Availability

The raw data supporting the conclusions of this article will be made available by the authors, without undue reservation.
